# Development of an integrated competency framework for postgraduate paediatric training: a Delphi study

**DOI:** 10.1007/s00431-021-04237-2

**Published:** 2021-09-08

**Authors:** Marieke Robbrecht, Koen Norga, Myriam Van Winckel, Martin Valcke, Mieke Embo

**Affiliations:** 1 Department of Internal Medicine and Pediatrics, Faculty of Medicine and Health Sciences, Ghent University, C. Heymanslaan 10, 9000 Ghent, Belgium; 2 Faculty of Medicine and Health Sciences, Antwerp University, Universiteitsplein 1, 2610 Wilrijk, Belgium; 3 Department of Paediatrics, Antwerp University Hospital, Drie Eikenstraat 655, 2650 Edegem, Belgium; 4 Department of Paediatrics, Ghent University Hospital, De Pintelaan 185, 9000 Ghent, Belgium; 5 Department of Educational Studies, Faculty of Psychology and Educational Sciences, Ghent University, H. Dunantlaan 2, 9000 Ghent, Belgium; 6 Expertise Network Health and Care, Artevelde University of Applied Sciences, Voetweg 66, 9000 Ghent, Belgium

**Keywords:** Paediatrics, Competency framework, Postgraduate training, Delphi methodology

## Abstract

Competency-based education (CBE) has transformed medical training during the last decades. In Flanders (Belgium), multiple competency frameworks are being used concurrently guiding paediatric postgraduate CBE. This study aimed to merge these frameworks into an integrated competency framework for postgraduate paediatric training. In a first phase, these frameworks were scrutinized and merged into one using the Canadian Medical Education Directives for Specialists (CanMEDS) framework as a comprehensive basis. Thereafter, the resulting unified competency framework was validated using a Delphi study with three consecutive rounds. All competencies (*n* = 95) were scored as relevant in the first round, and twelve competencies were adjusted in the second round. After the third round, all competencies were validated for inclusion. Nevertheless, differences in the setting in which a paediatrician may work make it difficult to apply a general framework, as not all competencies are equally relevant, applicable, or suitable for evaluation in every clinical setting. These challenges call for a clear description of the competencies to guide curriculum planning, and to provide a fitting workplace context and learning opportunities.

*Conclusion*: A competency framework for paediatric post-graduate training was developed by combining three existing frameworks, and was validated through a Delphi study. This competency framework can be used in setting the goals for workplace learning during paediatric training.

**Table:** 

**What is Known:** *•Benefits of competency-based education and its underlying competency frameworks have been described in the literature.* *•A single and comprehensive competency framework can facilitate training, assessment, and certification.*	
**What is New:** *•Three existing frameworks were merged into one integrated framework for paediatric postgraduate education, which was then adjusted and approved by an expert panel.* *•Differences in the working environment might explain how relevant a competency is perceived.*

**Supplementary information:**

The online version contains supplementary material available at 10.1007/s00431-021-04237-2.

## Introduction

During the last decades, competency-based education (CBE) has driven medical training towards the implementation of competency frameworks to evaluate clinical performance. Different general competency frameworks are available, such as the CanMEDS (Canadian Medical Education Directives for Specialists) Framework [1], the 6 core competencies of ACGME (the Accreditation Council for Graduate Medical Education) [2], and the Scottish Doctor [3]. Specific competency frameworks have also been developed for postgraduate paediatric training, such as the Curriculum for Common Trunk Training in Paediatrics [4] and The Pediatrics Milestones project [5].

CBE offers numerous benefits for a postgraduate paediatric training [6–10]. Its student-centred approach empowers students, facilitates goal-oriented self-directed learning, and stimulates learning within a limited timeframe [8, 11, 12]. It brings structure to the complex and unstructured clinical environment during workplace-based learning, the core of postgraduate medical education (PGME) [12, 13]. By providing explicit evaluation criteria, CBE ensures a more valid and objective assessment [7–11] as it emphasises accountability and transparency in medical education [9, 14]. CBE facilitates curriculum development [7, 8, 10], and it presents a utilitarian approach to curriculum planning, advocating that each curricular element should contribute to learner outcomes [6]. Moreover, CBE simplifies and supports the transition between education levels in medical curricula by guarantying learning continuity [9, 14]. Lastly, the focus on general competencies in CBE contributes to a holistic perspective of the medical profession [7–9].

Currently, different competency frameworks are alternately used in the paediatric training in Flanders (Belgium). First, the competency framework of the European Union of Medical Specialists (UEMS) [4] is very specific for the paediatric discipline. It consists of medical knowledge, technical skills, and general competencies. Although this framework is a guideline on how to become a competent paediatrician, it is only used as the basis for summative cognitive assessments and rarely for supporting workplace learning. Second, the Master of Specialistic Medicine (MSG) has defined four clusters (medical expert, scholar, communicator, manager) of generic competencies for all specialistic medicine disciplines, which are used for workplace-based assessment and certification. These clusters were extracted and adapted from the original CanMEDS framework, but this is not used in its original form during postgraduate training*.* In contrast, this original CanMEDS framework has dominantly been adopted in view of undergraduate training in Flanders and even has been validated in this setting [15]. Thus, the variability in adoption of these different competency frameworks hinders and complicates learning, assessment, and certification. The adoption of a unified and shared framework could enhance postgraduate paediatric training by ensuring coherence and continuity in evaluating clinical competence. Therefore, the present study aims at reporting the results of a validation study of an integrated competency framework for postgraduate paediatric training, after merging the UEMS, MSG, and CanMEDS frameworks.

## Materials and methods

### Constructing the competency framework

We developed a new integrated competency framework by combining 3 existing frameworks: the CanMEDS roles as defined by The Royal College of Physicians and Surgeons of Canada in 2015 [1], the ‘Curriculum for common trunk training in paediatrics’ as defined by UEMS [4] and the criteria as defined by MSG [16]. The CanMEDS framework was selected as the backbone framework because it is commonly accepted in Flemish undergraduate medical curricula and is already partially adopted in postgraduate medical education [13, 15, 17].

**Fig. 1:**
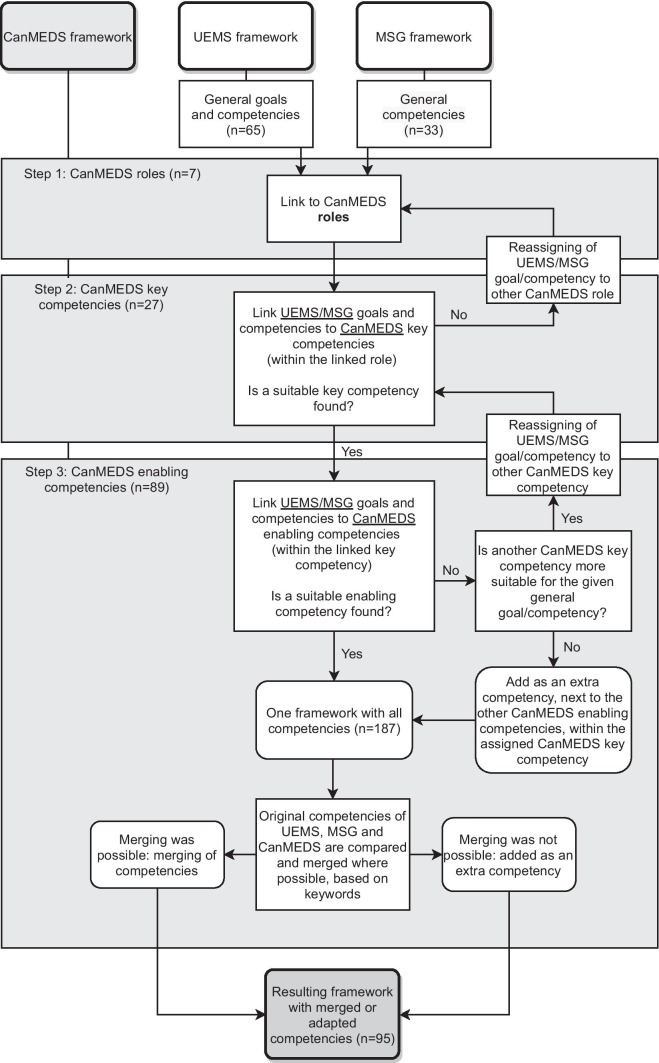
A flowchart of how the competency frameworks were merged

First, the main researcher (MR) linked the general goals and general competencies from the UEMS framework to the CanMEDS roles. Second, the goals and competencies from the UEMS framework were mapped on the key competencies linked to the CanMEDS roles. This version was reviewed by the research group (ME, MVW, VA, OJ, SVO). In a third step, each specific UEMS competency was linked to an enabling competency of the CanMEDS framework. This helped visualizing gaps and overlaps. These 3 steps were repeated for the MSG framework. Next, we looked for options to merge competencies based on keywords reflected in each competency in each of the three frameworks. When matching was impossible, the UEMS or MSG competencies were added to the CanMEDS competencies list. An overview of these different steps can be found in Fig. 1. All stages in the procedure were discussed with 2 other researchers (MVW, ME) until consensus was reached. All steps were documented in a Microsoft Excel® document to ensure methodological rigour. Two competencies, referring to discipline-specific knowledge and skills, were enriched with a list containing required specific paediatric knowledge and paediatric skills. In total, 65 competencies from the UEMS framework and 33 competencies from the MSG framework were linked to 89 enabling competencies of the CanMEDS framework. After the final stage in the procedure, researchers agreed on a baseline list of 95 competencies to be validated.

### Study design

The baseline framework was validated through an online survey using a Delphi methodology, which is a consensus method [18–20] regularly used to validate competencies [9]. Percentage agreement is common to define consensus in Delphi studies [21–24]. An agreement of 70% has been deemed to reflect a justifiable consensus level [19]. Building on the Likert-type scale scores, this meant that at least 70% of participants scored on either the positive or negative side of the Likert-type scale. For other questions, at least 70% of participants needed to answer either positively or negatively. Next to the analysis of the quantitative input, the qualitative data was analysed using inductive content analysis [25]. All analyses were performed in Microsoft Excel®.

The survey was piloted by KN and MVW, who are paediatricians, to check clarity and comprehensibility and to estimate time needed for completion. The piloting provided an indication of time required to complete the survey, and ensured clarity, reliability, and feasibility of the Delphi study [19, 26, 27].

### Participants

Purposive (non-probability) sampling was used to contact experts [20, 27, 28]. In order to ensure coverage across expertise domains [19, 26], participants were recruited from 5 different groups: recently graduated paediatricians, supervisors working as paediatricians in both general and university teaching hospitals in Flanders (Belgium), members of the accreditation committee of paediatrics in Belgium, educational experts with experience in medical education affiliated with Flemish Universities, and members of the paediatric section of the UEMS. No exclusion criteria were defined within these categories, as being related to one of our inclusion groups implied sufficient experience with paediatric postgraduate education. We initially aimed at 30 respondents, the ideal balance between decision quality and manageability of the data [19, 26, 27]*.* Participants were contacted via e-mail through the organisations to which they were affiliated. Participants were not anonymous to the researcher, but remained anonymous to each other [19]. Informed consent was obtained from each participant.

### Delphi process

The first Delphi round aimed to reach consensus regarding competency relevance for a graduating general paediatrician. A 6-point Likert-type scale (1 = not at all relevant to 6 = very relevant) was used by respondents, with the possibility to add comments. We used an even-numbered scale to encourage participants to think of a competency as either relevant or not for paediatric training [29].

After reaching consensus regarding relevance, the focus of the second round was to decide whether the competencies were clearly and appropriately formulated. Participating experts received the survey, supplemented with the level of consensus reached for each competency and the qualitative feedback from round one [19]. They were invited to comment on this input and to judge their relevance [20, 30, 31] using multiple choice questions. The third round focused on competencies that had not yet reached consensus in the previous rounds. These competencies were adjusted according to the feedback of experts. Participants were next asked to judge suitability for inclusion.

### Data collection

The online tool Qualtrics® was used to collect participants’ responses. A personal access link was sent by mail to each participant. Data was collected between August and December 2020 and stored on a secured Ghent University server. The study was conducted in English to prevent translation bias and to facilitate a follow-up study in other countries. However, participants could comment in their language of preference (Dutch, French, or English). To increase response rate, reminders were sent twice during each Delphi round to participants who had not (fully) completed the survey [27].

## Results

### Demographics

A total of 101 experts were contacted, of which 21 responded. In the first round, 11 (52.4%) experts completed the questionnaire. In the second round, 4 additional experts from the group of 21 initial responders were included who were not available in round 1. Although they did not participate in the first round, their inclusion was acceptable since the competency list did not change between the first and second round. In round 2, the survey was sent to these 15 participants, of which 13 (86.6%) completed the questionnaire. These 13 remaining experts all (100%) completed the survey in the third round. Demographics for participants who completed at least one round (*n* = 14) can be found in Table 1.

**Table 1:** Demographics of participants (*N* = 14)

Age	31–35 years old (*n* = 4) 36–40 years old (*n* = 1) 41–45 years old (*n* = 3) 51–55 years old (*n* = 4) 56–60 years old (*n* = 1) 61–65 years old (*n* = 1)
Functions*	Recently graduated as a paediatrician (2018 or later) (3) A member of medical education involved in competency-based education (3) Supervisor of paediatricians in training affiliated with a Belgian university (9) A member of the accreditation committee for paediatricians (2) A member of the paediatric section of UEMS (1)
University	K.U. Leuven (1) Antwerp University (4) Ghent University (9)
Supervised residents per year**	2 (2) 5 (1) 6 or more (7) None (not applicable) (2)

^*^Some participants had multiple functions, making the total amount greater than 14

^**^This information was not available for all 14 participants

### Survey flow

**Fig. 2:**
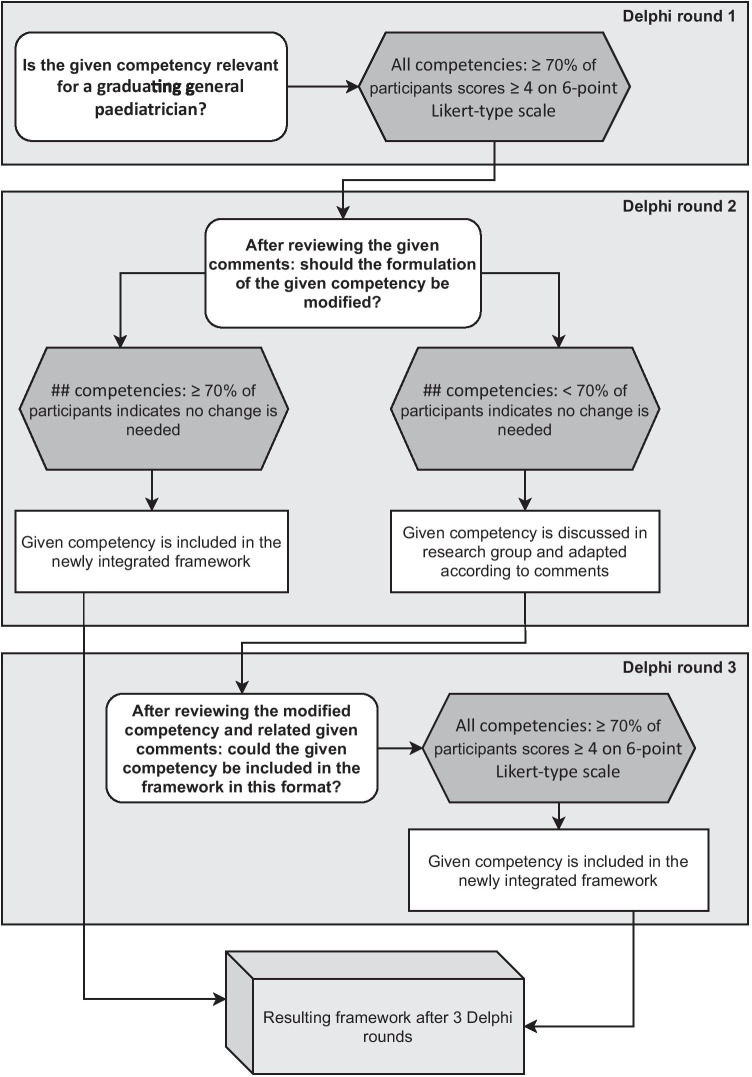
An overview of the survey flow in this Delphi study

An overview of the survey flow in this Delphi study can be found in Fig. 2.

#### First round

All competencies (*n* = 95) reached a positive 70% consensus as to their relevance. A majority (*n* = 69) reflected a 100% positive consensus. In total, 84 qualitative comments were given, that could be clustered into 4 areas: more applicable within a different role (*n* = 4), additional information from participants about their own scoring (*n* = 14), adjustments to the formulation (*n* = 26), and how the competencies could be acquired in the curriculum during workplace-based learning (*n* = 40).

#### Second round

Eighty-three competencies could be included as originally stated in round 1, leaving 12 competencies to be reformulated. One competency, ‘Perform the paediatric skills as listed in addendum, in a skilful and safe manner’, had comments regarding the corresponding skills list, but not regarding the competency itself. Adjustments were made in view of the roles of Medical Expert (*n* = 2), Communicator (*n* = 3), Leader (*n* = 4), Health Advocate (*n* = 2), and Scholar (*n* = 1). An overview of adjustments and adjustment rationales can be found in Table 2. Most suggestions for changes were related to the formulation not being specific enough for the paediatric profession or the wording being too vague. Other changes were related to concerns whether a competency was applicable for every general paediatrician, despite being scored as relevant in the first round. One example was ‘Contribute to the work of a research program’; comments questioned whether this is a prerequisite for being a good paediatrician.

**Table 2:** Overview of adjusted competencies

	**Competency before Delphi study**	**Adjusted competency after Delphi study**	**Reason for adjustment** t
1	Perform the paediatric skills as listed in addendum, in a skilful and safe manner (ADDENDUM: SAFE PRACTICAL SKILLS)	Perform the paediatric skills as listed in addendum, in a skilful and safe manner (ADJUSTED ADDENDUM: SAFE PRACTICAL SKILLS)	Not all skills in Addendum were relevant
2	Identify the limits of one’s own competency and act within them	Identify the limits of one’s own competency and act within them **by asking for help when needed**	‘Act within them’ unclear
3	Recognize when the values, biases, or perspectives of patients, physicians, or other healthcare professionals may have an impact on the quality of care, and modify the approach to the patient accordingly	**Consider an adapted approach in order to achieve the highest quality of care** when values, biases, or **(cultural)** perspectives of patients, physicians, or healthcare professionals **influence healthcare-related decisions**	Very broad, unclear
4	Respond to a patient’s non-verbal behaviours to enhance communication	Respond to a patient’s **and a patient’s caregivers** non-verbal behaviours to enhance communication	Not only patient’s, but also parents’ or other caregivers’ non-verbal behaviours
5	Adapt to the unique needs and preferences of each patient and to his or her clinical condition and circumstances, via effective communication and interpersonal skills in an age appropriate manner	Adapt to the unique needs and preferences of each patient and to his or her clinical condition and circumstances, via effective communication and interpersonal skills **adjusted to neurodevelopmental maturation**	Change to ‘adjusted to neurodevelopmental maturation’
6	Commit to quality assurance through systemic quality process evaluation and improvement	Commit to quality assurance **by taking into account** systemic quality process evaluation and improvement	No consensus regarding relevancy for every paediatrician
7	Improve the quality of patient care, by optimizing patient safety and maintenance of own expertise while using health informatics	Improve the quality of patient care, by optimizing patient safety and maintenance of own expertise while using health informatics **and other trustable information sources**	Not only health informatics can be used
8	Facilitate change in healthcare to enhance services and outcomes	Facilitate change in their own working environment and practice in order to ameliorate services and outcomes	No consensus regarding relevancy for every regional paediatrician
9	Participate in the organisation of health care and participate in representative functions within health care	Contribute to the organisation of health care **within their own facility**	No consensus regarding relevancy for every regional paediatrician
10	Use their influence and expertise in working with a community or population to identify the determinants of health that affect children in order to advance child health and well-being within their community	*Add the option: ‘Not applicable’*	No consensus regarding relevancy for every regional paediatrician
11	Identify the effects of local, national, and international policies on their work and contribute to a process to improve health in the community or population they serve	*Add the option: ‘Not applicable’*	No consensus regarding relevancy for every regional paediatrician
12	Contribute to the work of a research program (critical literature review, data collection and analysis, reporting research results)	*Some comments indicated that research is not relevant for every paediatrician. Nevertheless, this competency was scored as relevant in the first round and can therefore not easily be removed* *A proposal has been made to add ‘at least to some degree on individual basis’. However, we believe this makes the competency too open-ended and non-specific enough* *After careful consideration within our research team, we decided to not adjust this competency for two reasons* *1. As the post-graduate education requires a thesis for certification, every paediatrician should at least have conducted some kind of research once* *2. Although most peripheral paediatricians will not be conducting studies themselves, they might be confronted with the recruitment of participants and perhaps even data collection* *You can indicate whether further considerations are necessary*	No consensus regarding relevancy for every regional paediatrician

Not all 118 qualitative comments suggested to adjust formulation. Seven competencies were perceived as being dependent on the seniority of the resident. Three competencies were perceived as difficult to assess because direct observation influences the situation and thus assessment (*n* = 1), assessment of a competency can be very situational (*n* = 1), and it was unclear how to assess that particular competency (*n* = 1). Remaining individual comments addressed the need to train cultural competencies and to demonstrate a commitment to discuss mental health in physicians.

#### Third round

The 11 reformulated competencies all reached 100% consensus in the third and final round. The competency related to technical skills list was not reformulated, but as corresponding skills (*n* = 37) were tackled in the comments, the researchers included this list in the third round. However, validation of this list was out of the scope of this study, so no results are available. Nevertheless, it provided additional valuable information for e.g. Accreditation Committees. The final version of the validated competency framework is summarized in Table 3 (available online as supplementary material).

## Discussion

Three competency frameworks, currently used in Flemish postgraduate paediatric training, were merged into a single framework using a Delphi study. The integration of these different frameworks has been a meaningful exercise, and achieving consensus on this newly integrated framework from different stakeholders acknowledges the usefulness of this integration. By providing an integrated valid framework, the researchers aimed to support uniformity and clarity for clinical educators, professionals, and students in the context of self-directed learning during postgraduate training. Instead of using the former MSG framework, the results of the present study indicate advantages when using the integrated framework. A first advantage is that the new framework encompasses all seven CanMEDS roles. This ensures continuity throughout the medical training as these 7 CanMEDS roles are already being used during undergraduate training [15]. Furthermore, the integrated framework explicitly reflects a discipline-specific part in terms of knowledge and skills. This differs from the MSG competency framework that is often too broad to guide evaluation and feedback.

The general competencies were supplemented with a discipline-specific knowledge and skills lists. Both general and discipline-specific competencies are needed to become a competent paediatrician. Following this idea helps adopt a holistic curriculum perspective without focusing exclusively on discipline-specific competencies. This additional dimension might also help in supporting specific sub-disciplines within professions or addressing regional differences in responsibilities of paediatricians. Aside from the discipline specific knowledge and skills lists, the general framework is relevant to other medical specialist disciplines too, although the general competences might still differ in degree of urgency from one context to another and from discipline to discipline. Nevertheless, the approach reflected in the integrated framework prevents inconsistencies in how competencies are defined and developed [13]. Additionally, the integrated competency framework might support the general curriculum build-up, assessment and feedback practices, and certification of physicians [7–11, 14].

Although all competencies were scored as being relevant in the first round, comments surfaced during the second round regarding their relevance for every general paediatrician. Therefore, 2 competencies (see Table 2, competency 10 and 11) were labelled as ‘potentially not applicable’, pending the working and training settings for residents. Competency-based education focuses on the outcomes needed within the profession [7–10, 14], but paediatricians can work in many different settings, which might influence the contextual relevance of competencies. Nevertheless, it is important to uphold a standard in view of certification whereby further profiling may be an additional focus.

The relevance of one competency raised a particular debate. Several participants stressed that active participation in research should not be seen as a prerequisite for a paediatrician. This is in contrast to current training programs, in which a thesis and at least one publication are considered mandatory for graduation. The debate might result from a too ‘applied’ interpretation of competency-based education [6] that only looks at competencies that are considered directly applicable to professional activities.

Although the study aimed to validate the competency framework, caution should be taken to consider it as valid because as reflected in the comments, its implementation in a workplace-based learning curriculum should be further defined [9] and more input is needed to guide competency assessment [32]. As competencies evolve during training, attention should be paid to defining different levels of required competence for specific situations/settings, e.g. defining a short-term management plan for younger residents versus a long-term management plan for more advanced residents. To guide implementation and assessment in view of a required level of competence, supervisors—who are often not medical educators—will need a set of quality indicators to guide their training support [6]. Thus, the framework resulted by the Delphi study can be used by curriculum managers to review the curriculum. One possibility is to use the 5 steps of educational design, as described by Sherbino And Frank (2011): (1) needs assessment, (2) learning objectives, (3) instructional methods, (4) learner assessment, and (5) program evaluation. The curriculum review, based on the integrated competency framework and aforementioned steps, could improve the quality of learning, assessment, and certification of the competency framework within postgraduate training.

As professions evolve, the competency framework should also be considered as dynamic. This calls for a future follow-up of the current Delphi study. The starting point can now be the availability of a validated competency framework, based on a variety of views from multiple stakeholders. It offers a shared language and a professional standard. Next validation rounds will therefore be less time demanding and can start from the procedures and strategies outlined in the present Delphi study.

### Limitations

Although the researchers aimed at involving 30 participants, only 21 experts indicated initial willingness and only 14 completed at least one Delphi round. This might bias the results as consensus is easier to achieve within a smaller group. Nevertheless, the smaller group reflected multiple expertise fields and can be seen as a representative and qualitative group [33]. As the experts were contacted by email via professional organizations independent from our research network, it is possible that not all experts within our inclusion criteria were reached. Nevertheless, we emphasised the importance of the study to these organisations in view of improving future training programs. On the other hand, the increased workload for the participants because of the COVID-19 pandemic might also have affected their willingness or availability to participate, as time investment is a critical factor in a Delphi study [20].

New participants were also allowed to participate in the second round. Some might argue this could have affected consistency throughout the three rounds [19, 26]. However, we did not change the competency framework between the first and second rounds. Moreover, the new participants provided additional insightful comments, thus improving the quality of the competency framework.

Mainly experts affiliated with Flemish Universities were included, which might result in some bias due to localization. Nonetheless, since two international frameworks were used, namely the CanMEDS framework and the competency framework as established by the European Union of Medical Specialists (UEMS), the relevance of these frameworks supersedes the local setting. Though, future research should investigate the applicability of the validated framework in other countries.

Lastly, a real discussion between participants was not feasible, and additional questions to clarify comments could not be raised [18, 31]. Also, the process itself was time-consuming, which might have affected respondents’ commitment to the study.

## Conclusion

An integrated competency framework for postgraduate paediatric training was developed by combining three existing frameworks, using the CanMEDS framework as a basis, to provide a holistic view to the profession and supplemented with a discipline-specific knowledge and skills list. This integrated framework was validated through a Delphi study in view of its application in Flanders. Next steps will address curriculum planning in order to ensure competency assessment and development during workplace learning.

## Supplementary Information

Below is the link to the electronic supplementary material.

Supplementary file1 (DOCX 29 KB)

## Data Availability

The raw data are available upon request from the corresponding author.

## Abbreviations

ACGME: Accreditation Council for Graduate Medical Education
CanMEDS: Canadian Medical Education Directives for Specialists
CBE: Competency-based education
MSG: Master of Medicine in Specialist Medicine (“Master Specialistische Geneeskunde”)
PGME: Post-graduate medical education
UEMS: European Union of Medical Specialists
