# Supplementary material for: Development of an integrated competency framework for postgraduate paediatric training: a Delphi study
Source: Eur J Pediatr. 2021 Sep 8;181(2):637–46. doi: 10.1007/s00431-021-04237-2 (PMC8425852; doi:10.1007/s00431-021-04237-2)
Supplement: Supplementary file 1 — Supplementary file1 (DOCX 29 KB) [file 431_2021_4237_MOESM1_ESM.docx]

# Table 3: Competency framework for post-graduate paediatric training

| **Role 1: Medical expert** | | |
| --- | --- | --- |
|  | *Key competency 1: Practise medicine within their defined scope of practice and expertise* | |
|  |  | Apply knowledge of both the normal growth and development, as well as common and serious paediatric conditions as listed in addendum* (ADDENDUM: PAEDIATRIC EXPERTISE) |
|  |  | Perform appropriately timed clinical assessments with adequate responsiveness to situations where the wellbeing of the patient is endangered or compromised, and present recommendations in an organized manner |
|  |  | Carry out professional duties in the face of multiple, competing demands |
|  |  | Respond appropriately to the complexity, uncertainty, and ambiguity inherent in medical practice |
|  |  | Acknowledge the vulnerability of babies, children, and adolescents |
|  |  | Ensure the safeguarding of babies, children, and adolescents |
|  |  | Detect signs of problems concerning the wellbeing of babies, children, and adolescents |
|  | *Key competency 2: Perform a patient-centred clinical assessment and establish a management plan* | |
|  |  | Prioritize issues to be addressed in a patient encounter |
|  |  | Elicit a history and perform a physical examination for the purpose of formulating an appropriate (differential) diagnosis, management, disease prevention and health promotion |
|  |  | Establish goals of care in collaboration with patients and their families, which may include slowing disease progression, treating symptoms, achieving cure, improving function, and palliation |
|  |  | Establish a patient-centred management plan for common and serious paediatric conditions |
|  | *Key competency 3: Plan and perform procedures and therapies for the purpose of assessment and/or management* | |
|  |  | Order the appropriate investigations for paediatric assessment; interpret their results for the purpose of formulating an appropriate (differential) diagnosis; determine the most appropriate therapies or preventive interventions including the safe prescription of common drugs; all in an evidence-based manner |
|  |  | Obtain and document informed consent, with correct explanation of the risks and benefits of, and the rationale for, a proposed procedure or therapy |
|  |  | Prioritize a procedure or therapy, taking into account clinical urgency and available resources |
|  |  | Perform the paediatric skills as listed in addendum, in a skilful and safe manner (ADDENDUM: SAFE PRACTICAL SKILLS (ADAPTED)) |
|  | *Key competency 4: Establish plans for ongoing care and, when appropriate, timely consultation* | |
|  |  | Implement a patient-centred care plan that supports ongoing care |
|  |  | Follow-up on investigations, response to treatment, and further consultation in the management of acute or chronic illness in children |
|  | *Key competency 5:* Actively contribute, as an individual and as a member of a team providing care, to the continuous improvement of health care quality and patient safety | |
|  |  | Recognize harm from healthcare delivery, including patient safety incidents; and display a response to it |
|  |  | Identify the limits of one's own competency and act within them by asking for help when needed |
|  |  | Deliver the highest quality of care, including the adoption of strategies and the participation in activities that contribute to the promotion of patient safety, and address human and system factors |
| **Role 2: Communicator** | | |
|  | *Key competency 1: Establish professional therapeutic relationships with patients and their*  *families* | |
|  |  | Communicate correctly and efficiently, using a patient-centred approach that encourages patient trust and autonomy and is characterized by empathy, respect, sensitivity and compassion to establish a positive therapeutic relationship with patients and their families |
|  |  | Optimize the physical environment for patient comfort, dignity, privacy, engagement, and safety |
|  |  | Consider an adapted approach in order to achieve the highest quality of care when values, biases or (cultural) perspectives of patients, physicians or healthcare professionals influence healthcare related decisions |
|  |  | Respond to a patient's and a patient's caregivers non-verbal behaviours to enhance communication |
|  |  | Manage disagreements and emotionally charged conversations |
|  |  | Adapt to the unique needs and preferences of each patient and to his or her clinical condition and circumstances, via effective communication and interpersonal skills adjusted to neurodevelopmental maturation |
|  | *Key competency 2: Elicit and synthesize accurate and relevant information, incorporating the perspectives of patients and their families* | |
|  |  | Use patient-centred interviewing skills and active listening skills to effectively elicit and draw together relevant biomedical and psychosocial information and perspectives |
|  |  | Provide a clear structure for and manage the flow of an entire patient encounter |
|  |  | Seek and synthesize relevant information and perspectives from other sources, including the patient's family or other healthcare professionals, with patient's consent |
|  | *Key competency 3: Share health care information and plans with patients and their families* | |
|  |  | Communicate relevant understandable oral and written information and explanations to (young) patients and their families that are clear, accurate, and timely, while checking for patient and family understanding |
|  |  | Communicate bad news to (young) patients and their families in a clear, accurate and respectful manner and provide support in a crisis situation |
|  |  | Disclose harmful patient safety incidents to patients and their families accurately and appropriately |
|  | *Key competency 4: Engage patients and their families in developing plans that reflect the patient’s health care needs and goals* | |
|  |  | Facilitate discussions with patients and their families in a way that is respectful, non-judgmental, and culturally safe on issues, problems and plans to develop a shared plan of care |
|  |  | Support patients and their families to identify, access, and make use of information and communication technologies to support their care and manage their health |
|  |  | Use communication skills and strategies that help patients and their families make informed decisions regarding their health |
|  | *Key competency 5: Document and share written and electronic information about the medical encounter to optimize clinical decision-making, patient safety, confidentiality, and privacy* | |
|  |  | Document clinical encounters in medical (hospital) records and legal documents in an accurate, comprehensive, complete, timely, accessible manner, in compliance with regulatory and legal requirements |
|  |  | Communicate effectively by clear record-keeping and report-writing using a written health record, electronic medical record, or other digital technology |
|  |  | Share information with patients and others in a manner that respects patient privacy, confidentiality, and autonomy and enhances understanding and ability to consent |
| **Role 3: Collaborator** | | |
|  | *Key competency 1: Work effectively with physicians and other colleagues in the healthcare professions* | |
|  |  | Demonstrate efficient and effective communication and interpersonal skills for the establishment of positive relationships with physicians and other colleagues in the healthcare professions that support relationship-centered collaborative care |
|  |  | Negotiate overlapping and shared responsibilities with physicians and other colleagues in the health care professions in episodic and ongoing care |
|  |  | Participate appropriately in a professional healthcare team, including the engagement in respectful shared decision-making, to achieve optimal patient care |
|  | *Key competency 2: Work with physicians and other colleagues in the health care professions to promote understanding, manage differences, and resolve conflicts* | |
|  |  | Show professional respect for the views and contributions of colleagues in a range of roles in paediatric practice. |
|  |  | Implement strategies to promote understanding, manage differences, and resolve conflicts in a manner that supports a collaborative culture |
|  | *Key competency 3: Hand over the care of a patient to another health care professional to facilitate continuity of safe patient care* | |
|  |  | Determine when and to whom additional advice, opinion, help, support or supervision should be asked for and care should be transferred to another physician or healthcare professional |
|  |  | Demonstrate safe handover, referral and discharge planning of care; using both verbal and written communication during a patient transition to a different health care professional, setting or stage of care |
| **Role 4: Leader** | | |
|  | *Key competency 1: Contribute to the improvement of health care delivery in teams, organizations, and systems* | |
|  |  | Commit to quality assurance by taking into account systemic quality process evaluation and improvement |
|  |  | Contribute to the organisation of health care within their own facility |
|  |  | Contribute to a culture that promotes patient safety |
|  |  | Analyse patient safety incidents to enhance systems of care |
|  |  | Improve the quality of patient care, by optimizing patient safety and maintenance of own expertise while using health informatics and other trustable information sources |
|  | *Key competency 2: Engage in the stewardship of health care resources* | |
|  |  | Allocate healthcare resources for optimal patient care |
|  |  | Apply evidence and management processes to achieve cost-appropriate care |
|  | *Key competency 3: Demonstrate leadership in professional practice* | |
|  |  | Demonstrate leadership skills by effectively assign, delegate and follow-up on tasks to enhance healthcare |
|  |  | Manage stressful situations with effective responses to challenge, complexity and stress in paediatrics |
|  |  | Facilitate change in their own working environment and practice in order to ameliorate services and outcomes |
|  | *Key competency 4: Manage career planning, finances, and health human resources in a practice* | |
|  |  | Set priorities and maintain effective time management skills to integrate practice and personal life |
|  |  | Manage a career and a practice |
|  |  | Implement processes to ensure personal practice improvement |
| **Role 5: Health advocate** | | |
|  | *Key competency 1: Respond to an individual patient’s health needs by advocating with the patient within and beyond the clinical environment* | |
|  |  | Work with patients to address determinants of health that affect them and their access to needed health services or resources |
|  |  | Use their influence and expertise to increase opportunities for patients and their families to adopt healthy behaviours, and advance health as well as the well-being of individual patients and their families |
|  |  | Incorporate disease prevention, health promotion, and health surveillance into interactions with individual patients |
|  | *Key competency 2: Respond to the needs of the communities or populations they serve by advocating with them for system-level change in a socially accountable manner* | |
|  |  | Use their influence and expertise in working with a community or population to identify the determinants of health that affect children in order to advance child health and well-being within their community (if applicable) |
|  |  | Improve clinical practice by applying a process of continuous quality improvement to disease prevention, health promotion, public health issues and health surveillance activities |
|  |  | Identify the effects of local, national and international policies on their work and contribute to a process to improve health in the community or population they serve (if applicable) |
| **Role 6: Scholar** | | |
|  | *Key competency 1: Engage in the continuous enhancement of their professional activities through ongoing learning* | |
|  |  | Make a lifelong commitment to learning by accepting responsibility for developing, implementing, monitoring and revising a personal continuing education strategy to enhance professional practice |
|  |  | Regularly reflect on and assess their performance using various internal and external data sources to identify opportunities for learning and improvement by holding a positive approach to receiving mentoring and educational supervision |
|  |  | Engage in collaborative learning to continuously improve personal practice and contribute to collective improvements in practice |
|  | *Key competency 2: Teach students, residents, the public, and other health care professionals* | |
|  |  | Demonstrate effective teaching, with the recognition of the influence of role-modelling and the impact of the formal, informal, and hidden curriculum on learners |
|  |  | Promote a safe learning environment |
|  |  | Ensure patient safety is maintained when learners are involved |
|  |  | Plan and deliver a learning activity to students, colleagues and other healthcare professionals |
|  |  | Provide feedback to enhance learning and performance |
|  |  | Assess and evaluate learners, teachers, and programs in an educationally appropriate manner |
|  | *Key competency 3: Integrate best available evidence into practice* | |
|  |  | Generate focused questions that address practice uncertainty and knowledge gaps in clinical and other professional encounters |
|  |  | Identify, select and navigate pre-appraised research resources such as publications and electronic literature databases |
|  |  | Critically evaluate the integrity, reliability, and applicability of health related research and literature |
|  |  | Integrate evidence into decision-making in their practice |
|  | *Key competency 4: Contribute to the creation and dissemination of knowledge and practices applicable to health* | |
|  |  | Demonstrate an understanding of the scientific principles of research and scholarly inquiry and the role of research evidence in healthcare |
|  |  | Recognize special issues pertaining to children participating in research, and identify ethical principles for research and incorporate them into obtaining informed consent, considering potential harms and benefits, and considering vulnerable populations |
|  |  | Contribute to the work of a research program (critical literature review, data collection and analysis, reporting research results) |
|  |  | Pose questions amenable to scholarly inquiry and select appropriate methods to address them |
|  |  | Summarize and communicate to professional and lay audiences, including patients and their families, the findings of relevant research and scholarly inquiry |
| **Role 7: Professional** | | |
|  | *Key competency 1: Demonstrate a commitment to patients by applying best practices and adhering to high ethical standards* | |
|  |  | Exhibit appropriate professional behaviours and relationships in all aspects of practice, demonstrating honesty, integrity, humility, commitment, compassion, respect, altruism, respect for diversity, and maintenance of confidentiality. |
|  |  | Demonstrate a commitment to excellence in all aspects of practice |
|  |  | Demonstrate reliability and responsibility in continuity of care by ensuring their accessibility to colleagues, patients and their families |
|  |  | Demonstrate ethical personal and professional practice, including recognizing and responding to ethical issues encountered in practice and showing sensitivity and responsiveness to a diverse patient population |
|  |  | Manage conflicts of interest while following the principle that all decisions are to be made in the best interests of the patient |
|  |  | Exhibit professional behaviours in the use of technology-enabled communication |
|  | *Key competency 2: Demonstrate a commitment to society by recognizing and responding to societal expectations in health care* | |
|  |  | Demonstrate accountability to patients, society, and the profession by responding to societal expectations of physicians |
|  | *Key competency 3: Demonstrate a commitment to the profession by adhering to standards and participating in physician-led regulation* | |
|  |  | Fulfil and adhere to the professional and ethical codes, standards of practice, laws governing practice and comply with all legal and moral obligations for reporting disease and potential or real abuse/neglect |
|  |  | Recognize and respond to unprofessional and unethical behaviours in physicians and other colleagues in the healthcare professions |
|  |  | Participate in peer assessment and standard-setting |
|  | *Key competency 4: Demonstrate a commitment to physician health and well-being to foster optimal patient care* | |
|  |  | Exhibit self-awareness and a responsible approach to the health, stress, well-being and professional performance of their own |
|  |  | Manage personal and professional demands for a sustainable practice throughout the physician life cycle, and to manage personal demands in their accessibility to colleagues, patients and their families |
|  |  | Maintain the health of the team they work with and promote a culture that recognizes, supports and responds effectively to colleagues in need |

*The competency framework consists of 7 roles. Each role has corresponding key competencies, which are subdivided in in enabling competencies.*
